# Development of flow cytometric opsonophagocytosis and antibody-mediated complement deposition assays for non-typeable *Haemophilus influenzae*

**DOI:** 10.1186/s12866-018-1314-5

**Published:** 2018-10-29

**Authors:** Stephen R. Thomas, Stephanie Leung, Katy Knox, Tom M. A. Wilkinson, Karl J. Staples, Pascal Lestrate, Dominique Wauters, Andrew Gorringe, Stephen C. Taylor

**Affiliations:** 1 Public Health England, Microbiological Services, Porton Down, Salisbury, SP4 0JG UK; 2 Clinical & Experimental Sciences, University of Southampton Faculty of Medicine, Southampton General Hospital, Tremona Road, Southampton, UK; 3 GSK, Rixensart, Belgium

**Keywords:** Non-typeable *Haemophilus influenzae*, Antibody, Opsonophagocytosis, Complement, Flow cytometry, Vaccine

## Abstract

**Background:**

*Haemophilus influenzae* is found in the nasopharynx of 80% of the human population*.* While colonisation with non-typeable *Haemophilus influenzae* (NTHi) is usually asymptomatic, it is capable of causing acute and chronic otitis media (OM) in infants, invasive disease in susceptible groups and is the leading cause of exacerbations of patients with chronic obstructive pulmonary disease (COPD).

Current methods for assessing functional antibody immunity to NTHi are limited and labour intensive. Flow cytometric assays could provide an attractive alternative to evaluate immune responses to candidate vaccines in clinical trials.

**Results:**

We have developed a duplexed flow-cytometric uptake and oxidative burst opsonophagocytosis assay (fOPA). We have also developed a duplexed antibody-mediated complement C3b/iC3b and C5b-9 deposition assay (CDA). Antibody-mediated C3b/iC3b deposition correlated with opsonophagocytic uptake (*r* = 0.65) and with opsonophagocytic oxidative burst (*r* = 0.69). Both fOPA and CDA were reproducible, with the majority of samples giving a coefficient of variation (CV) of < 20% and overall assay CVs of 14% and 16% respectively.

**Conclusions:**

The high-throughput flow cytometric assays developed here were successfully optimised for use with NTHi. Assays proved to be sensitive and highly reproducible for the measurement of bacterial uptake and oxidative burst opsonophagocytosis and antibody-mediated deposition of C3b/iC3b and C5b-9. These assays are useful tools for use in large scale epidemiological studies and to assist in the assessment of functional antibody induced by NTHi candidate vaccines.

**Electronic supplementary material:**

The online version of this article (10.1186/s12866-018-1314-5) contains supplementary material, which is available to authorized users.

## Background

*Haemophilus influenzae* are common colonising organisms of the human nasopharynx, found in 80% of the human population [1]. The majority of colonisation is made up of unencapsulated or non-typeable *H. influenzae* (NTHi) strains [1]. Although colonisation with NTHi is usually asymptomatic, it is also capable of causing disease, accounting for approximately 20–40% of all cases of acute and recurrent acute otitis media (AOM) infections in young children [2–4]. More worrying is the impact of NTHi infection as a cause of exacerbations in chronic obstructive pulmonary disease (COPD) patients, with 25–80% of cases resulting in severe respiratory complications [5]. In the UK alone, 1 million people are diagnosed with COPD while a further 2 million are estimated to be undiagnosed [6]. Moreover, at any time 30% of COPD patients are colonised with NTHi [7]. The incidence of invasive disease, such as septicaemia, pneumonia and meningitis as a result of NTHi infection, although still relatively rare, has also been observed in specific risk groups and has been increasing in prevalence over the last two decades [8].

Prevention of disease and concerns of the possible emergence of antibiotic resistance due to repeated and inappropriate treatment is becoming a high priority, and a vaccine to protect against NTHi disease would be of particular value [9]. The lack of a capsule has meant that the search for a vaccine has concentrated on identifying suitable outer membrane proteins [10]. To date there are a number of conserved outer membrane proteins that have been identified as possible vaccine candidates [9–11], one of which has been used as a carrier protein in GSK’s 10-valent pneumococcal conjugate vaccine [12]. Studies have shown a reduction in the incidence of OM in children due to pneumococcal infection and also NTHi [13–15]. Previous studies have developed serum bactericidal assays (SBA) [16] or killing opsonophagocytosis assays (kOPA) [17] to measure functional antibody-mediated immunity to NTHi. However, while SBA has been established as a correlate of protection for invasive disease caused by encapsulated *Haemophilus influenzae* type b (Hib) and has been used in efficacy studies for Hib vaccines [18] a reliable correlate of protection has yet to be identified for disease due to NTHi (e.g. AOM, exacerbation of COPD) [19]. A human challenge model showed that colonised individuals showed a 4-fold increase in serum levels of IgA, IgM or IgG [20]. Modest bactericidal activity has been observed against homologous NTHi strains in convalescent sera of children with a previous AOM infection [21], with further smaller studies showing bactericidal activity to the homologous strain lacking in acute sera but present in convalescent sera which appears not to induce protection from heterologous strains [3, 22, 23]. However, a large natural immunity study or vaccine efficacy study has yet to be carried out in order to establish SBA as an immune correlate of protection for NTHi disease. Both assays could result in reproducible methods that would only require minimal volumes of sera and could greatly enhance candidate vaccine testing.

Antibody-mediated deposition of C3b and C5b-9 is required for opsonophagocytosis and bactericidal activity respectively, thus analysis of the antibody-mediated deposition of these complement components could inform the analysis of immune responses to NTHi natural infection and vaccines.

## Materials and methods

### Serum samples

Pre-and post-vaccination serum (*n* = 6 and 7 respectively) were convenience samples that had been previously collected from healthy laboratory staff based at Public Health England (PHE), Porton Down, following a single dose of *Synflorix.* Vaccination had been offered to staff working in laboratories using cultures of NTHi and *Streptococcus pneumoniae*. All sera were collected in 8.5 ml serum separation tubes (BD vacutainer SST advance gold), heat-inactivated for 30 min at 56 °C and then stored at 4 °C for short-term storage, or − 20 °C for long-term storage. Sera raised in mice immunised with heat-killed NTHi bacteria formulated with 0.33% Alhydrogel at days 0, 21, and 28 with a terminal bleed at day 35 were used, as well as a mouse non-immune serum (NIS) obtained using Alhydrogel only as a negative control. Sera (*n* = 35) were obtained from subjects with stable mild and moderate COPD (provided by University of Southampton Faculty of Medicine).

### Complement source

IgG-depleted human plasma from healthy volunteers was prepared as described by Brookes et al. [24] and used for all assays. The complete removal of IgG from the preparation was confirmed by IgG ELISA (Bethyl Laboratories), performed according to manufacturer’s instructions and showed values below the level of detection.

### Bacteria

NTHi strains 3655, 3224A and MPJ003 (supplied by GSK) were grown on chocolate agar, supplemented with PolyVitex (Biomerieux SA, France), overnight at 37 °C in 5% CO_2_. The growth from each plate was re-suspended in 2.5 ml brain heart infusion broth supplemented with hemin and nicotinamide adenine dinucleotide (NAD) (sBHI). The OD_620nm_ was determined for the 2.5 ml culture, which was then used to calculate the volume of inoculum required to give a starting OD_620nm_ of 0.08 in sBHI. Cultures were incubated at 37 °C with shaking at 180 rpm until an OD_620nm_ of 0.35–0.45 was achieved (mid-log phase). For use in all other assays, other than the flow cytometric OP assay, bacteria were then washed in 1 ml phosphate-buffered saline (PBS) to create a working stock. For use in the flow cytometric OP assay bacteria were fluorescently labelled with a 1 mM stock solution of CellTrace violet cell proliferation kit (Life technologies) prepared by adding dimethyl sulphoxide (DMSO) to the appropriate number of vials to give a final working concentration of 100 μM. Bacteria were incubated for 20 min at 37 °C with shaking at 180 rpm and protected from light. Bacteria were washed once in PBS before re-suspending the pellets in 1 ml PBS. The bacterial concentration was calculated assuming that an OD_620_ of 1.0 corresponds to 4.0 × 10^9^ml^− 1^ colony forming units.

### Cell line growth and differentiation

HL-60 cells (human promyelocytic leukemia cells; CCL240; American Type Culture Collection, Rockville, USA) were maintained and differentiated into granulocytes as described by Humphries et al. [25].

### Duplexed uptake and oxidative burst flow-cytometric opsonophagocytosis assay (fOPA)

CellTrace violet-labelled bacteria were prepared at 5.0 × 10^9^ ml^− 1^ in blocking buffer (OPA-BB) (2% skimmed milk powder in HBSS containing 1.2 mM CaCl_2_.2H_2_O and 1 mM MgSO_4_.7H_2_O). 5 μl of heat inactivated serum was added to appropriate wells of a standard U-bottom 96-well plate. 15 μl of OPA-BB was added to wells containing sera, or an appropriate amount to control wells to give a final volume of 40 μl prior to addition of HL-60 cells. 10 μl bacteria were added to every well except the HL-60 cells-only control. Samples were incubated for 15 min at 37 °C with shaking at 900 rpm. 10 μl of 1:10 diluted IgG-depleted human plasma (diluted in OPA-BB) was added to appropriate wells, followed by a further incubation for 7.5 min at 37 °C with shaking at 900 rpm. Differentiated HL-60 cells at 2.5 × 10^7^ ml^− 1^ were prepared in OPA-BB and 25 μl added to all wells along with a further 25 μl Dihydrorhodamine 123 (25 μg/ml DHR 123 – Life Technologies), and incubated for 15 min at 37 °C with shaking at 900 rpm. Following this, microtitre plates were immediately placed on ice and samples fixed with 80 μl 1% formaldehyde in Dulbecco’s PBS + 0.02% (*w*/*v*) EDTA and incubated for 30 min at RT in the dark before analysis by flow cytometry. All tests were performed in duplicate and the following controls were used: HL-60 cells only (unstained), HL-60 cells only (DHR-123 stained), HL-60 cells plus bacteria and finally HL-60 cells plus bacteria and complement. Two wells containing 10 μl phagotest (Glycotope Biotechnology), with 20 μl buffer, 10 μl 5% complement and 50 μl HL-60 cells were also included. The phagotest (*Escherichia coli)* is pre-stained with a FITC stain and pre-opsonised therefore no stain or serum was added.

### Flow-cytometric complement C3b/iC3b and C5b-9 deposition assay (CDA)

5 μl heat-inactivated test sera were added to the relevant wells of a standard U-bottom 96-well microtitre plate, followed by 2 μl IgG-depleted human plasma and 93 μl bacteria at an OD_620nm_ 0.1 in CDA-BB (2% bovine serum albumin in PBS *w*/*v*). The plate was then incubated at 37 °C for 45 min with shaking at 900 rpm. Following incubation, the plate was centrifuged at 3060 g for 5 min and washed using CDA-BB. The resulting pellet was re-suspended in 200 μl of conjugate (FITC-conjugated rabbit polyclonal antibody to human C3c (Abcam, UK) and murine sC5b-9 neoantigen monoclonal antibody (Quidel, US, clone 056B-75.2.3.10) custom conjugated to Alexafluor 647 (Life Technologies Ltd., UK)) which were used at a 1:500 and 1:4000 dilution respectively. The plate was then incubated for a further 20 min at 4 °C. Following this the plate was centrifuged at 3060 g for 5 min and washed with CDA-BB; this wash step was repeated twice more before the pellet was finally re-suspended with 1% formaldehyde in PBS and incubated for 30 min at RT. The completed assay was analysed by flow cytometry on the same day. All tests were performed in duplicate and the following controls were used in each assay: bacteria only, bacteria and detection antibody only and finally bacteria with complement and conjugate only.

### Flow cytometric analyses

Samples were analysed using a Beckman Coulter Cyan flow cytometer equipped with a Cytek 96-well microtitre plate sampler. Protocols were established to analyse profiles of events identified on the cytometer by the forward scatter (FS), measuring the size of the cell, and side scatter (SS), measuring the granularity and internal structural complexity. For each sample, approximately 10,000 individual events were analysed for fluorescence and a horizontal gate was drawn to include 10% of the antibody-independent control sample population (bacteria plus IgG-depleted human plasma plus conjugate for CDA and HL-60 cells plus bacteria plus IgG-depleted plasma for fOPA). A mean fluorescence index (MFI) was calculated for each sample, which involved the multiplication of the percent of events moving into the horizontal gate (%-gated), by the average fluorescence of that population (X-mean). The final result for each test was expressed as the average MFI (average FI taken for duplicate test samples) of the test serum sample minus the average MFI of the antibody-independent control sample population (MFI-control).

## Results

A fOPA was developed from protocols previously described for *Neisseria meningitidis* by Findlow et al. 2006 and Humphries et al. 2015 [25, 26]. Early results showed either low or variable levels of opsonophagocytosis, with high antibody-independent fluorescence masking antibody-mediated opsonophagocytosis, therefore optimisation was required.

The opsonophagocytic uptake protocol used by Findlow for *N. meningitidis* [26] had two 7.5 min incubation steps, the first following addition of serum, bacteria and complement and the second following addition of differentiated HL-60 cells. However when this approach was used with NTHi the results showed high levels of antibody-independent fluorescence and therefore very little distinction was observed for antibody-mediated uptake of bacteria (Additional file 1: Figure S1). The assay was therefore performed in 3 steps (30 min with serum plus bacteria, 15 min following addition of complement and finally 30 min following addition of differentiated HL-60 cells) [25]. Using this protocol increased antibody-mediated fluorescence compared to the no antibody control values was observed (Additional file 2: Figure S2A). These times were reduced to 15 min, 7.5 min and 15 min when duplexing the fOPA to include measurement of oxidative burst with no loss of assay sensitivity (Additional file 2: Figure S2B).

**Fig. 1:**
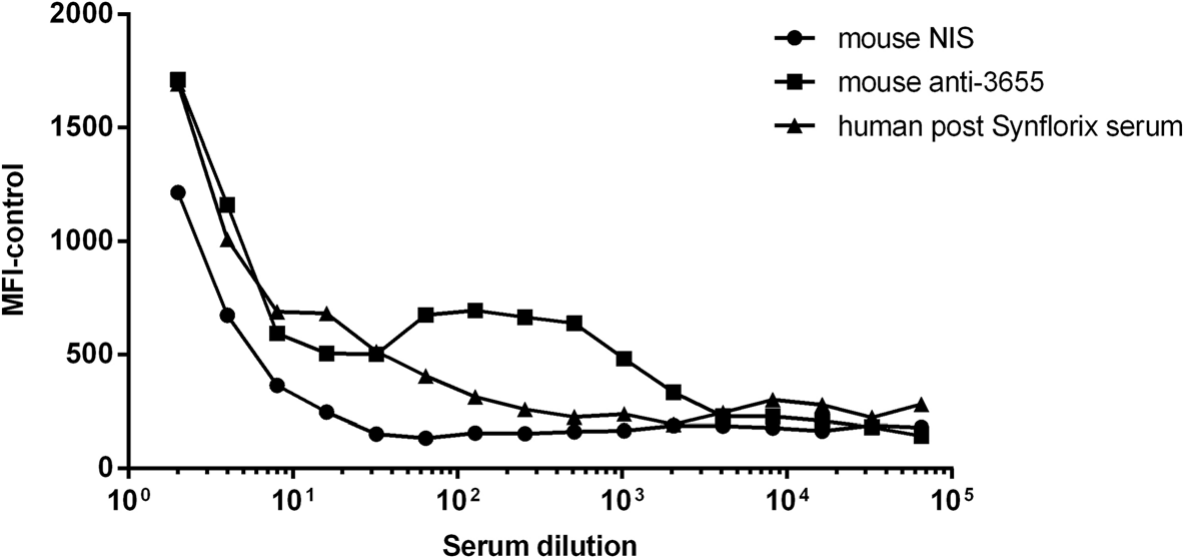
Selection of a fluorescent dye to measure bacterial uptake (fOPA). CellTrace violet-labelled NTHi strain 3655 was incubated with IgG-depleted plasma, differentiated HL60 cells and dilutions of mouse non-immune (circles), mouse anti-3655 whole bacteria (squares) or human post *Synflorix* (triangles) serum. Each point is the mean of duplicate samples

Two fluorochromes were evaluated for staining of the bacteria to investigate phagocytic uptake. Live NTHi were incubated with BCECF, AM (2′,7′-*bis*-(2-carboxyethyl)-5-(and-6)-carboxyfluorescein acetoxymethyl ester) (described by Humphries et al.) [25] or CellTrace™ violet cell proliferation dye (CTV). Unexpectedly, BCECF staining of NTHi appeared to inhibit uptake by HL-60 cells and was therefore unsuitable for the assay (Additional file 3: Figure S3). However, the same assay carried out with CTV-stained NTHi showed a serial dilution of OPA activity mediated by antibody in post-Synflorix human serum (Fig. 1). While the mouse NIS and mouse anti-3655 sera were initially used to provide a negative and positive control, the dilution data also showed that there was a clear increase following administration of the NTHi whole cell vaccine. Thus CTV-stained NTHi were used to determine uptake by HL-60 cells and the assay serum concentration was established.

**Fig. 2:**
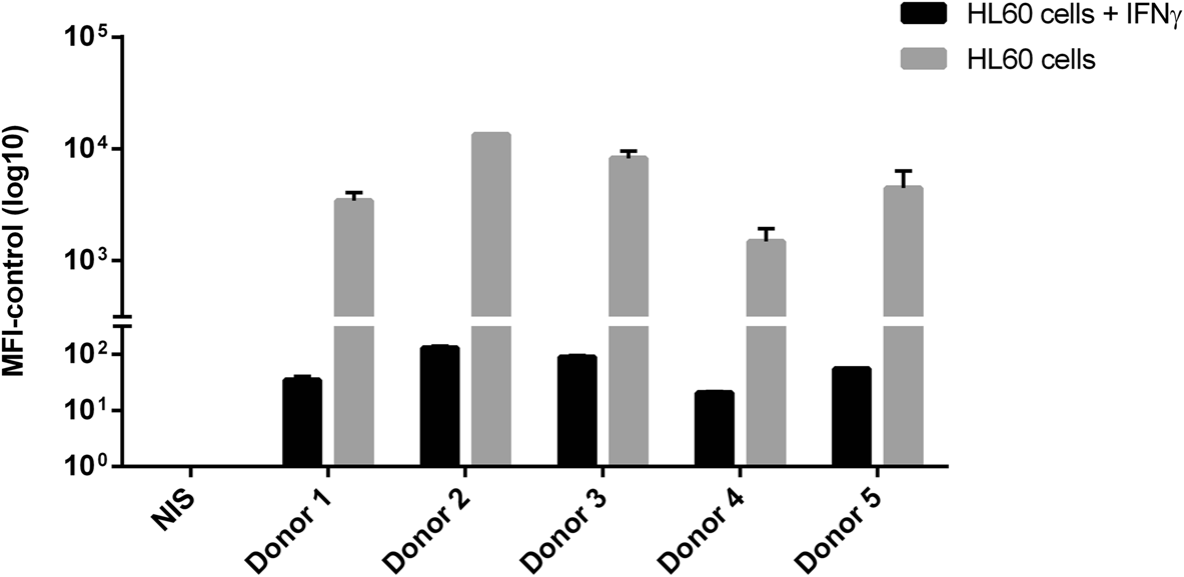
Assessing the effects of pre-treatment of HL60 cells with IFNƴ on oxidative burst. NTHi strain 3224A was incubated with differentiated HL60 cells in the presence or absence of IFNƴ and either mouse non-immune serum (NIS) or human post *Synflorix* sera. Error bars represent the standard deviation of duplicate samples

In addition to using fluorescently-labelled bacteria to measure opsonophagocytic uptake, a fluorescent stain to detect oxidative burst was also investigated. Dihydrorhodamine 123 (DHR-123) has been used in a number of previously described opsonophagocytosis assays [27, 28]. These experiments were performed using HL-60 cells pre-stimulated with IFNƴ, as this has previously been shown to improve oxidative burst [29]. However, the omission of IFNƴ resulted in considerably higher MFI-control values when compared to those with pre-stimulation (Fig. 2). Initially oxidative burst by differentiated HL-60 cells was evaluated independently of uptake. Oxidative burst by the HL-60 cells in the presence of NTHi and human serum was considerably lower than seen previously with *N. meningitidis* [25] (Fig. 2: Black bars).

Parallel measurement of both uptake of bacteria and oxidative burst was then assessed using a panel of 17 sera. Correlations between duplexed uptake and oxidative burst fluorescence for strains 3655, 3224A and MPJ003 were 0.85, 0.89 and 0.97 respectively (*p* = < 0.001). Additional file 4: Figure S4 shows the relationship between opsonophagocytic uptake and oxidative burst for individual sera with NTHi, strain 3224A (Additional file 4: Figure S4). Correlations between the duplexed assay and a single parameter OPA uptake assay for strains 3655, 3224A and MPJ003 were 0.74, 0.79 and 0.94 respectively (*p* = < 0.001). Between the duplexed assay and single parameter OPA oxidative burst assay, correlations for strains 3655, 3224A and MPJ003 were 0.67 (*p* = 0.003), 0.71 (*p* = 0.002) and 0.97 (*p* = < 0.001) respectively. All correlations are expressed as r values by Pearson correlation coefficient. Inter-assay precision of the duplexed assay was determined by one operator over 3 days for strains 3224A and 3655, and over 4 days for strain MPJ003. Low CV values were observed for all assays with the majority of samples giving a CV of < 20% for uptake and oxidative burst (Table 1).

**Table 1:** OPA-uptake and OPA**-** oxidative burst inter-assay variability for strains 3655, 3224A and MPJ003 (17 sera per strain in triplicate, *n* = 153)

Assay	Sera with defined CV				Mean CV (±SEM)
	< 20%	< 35%	< 40%	> 40%	
OPA – uptake	42	8	1	0	13.20 (1.10)
OPA – oxidative burst	40	8	2	1	14.63 (1.19)

**Fig. 3:**
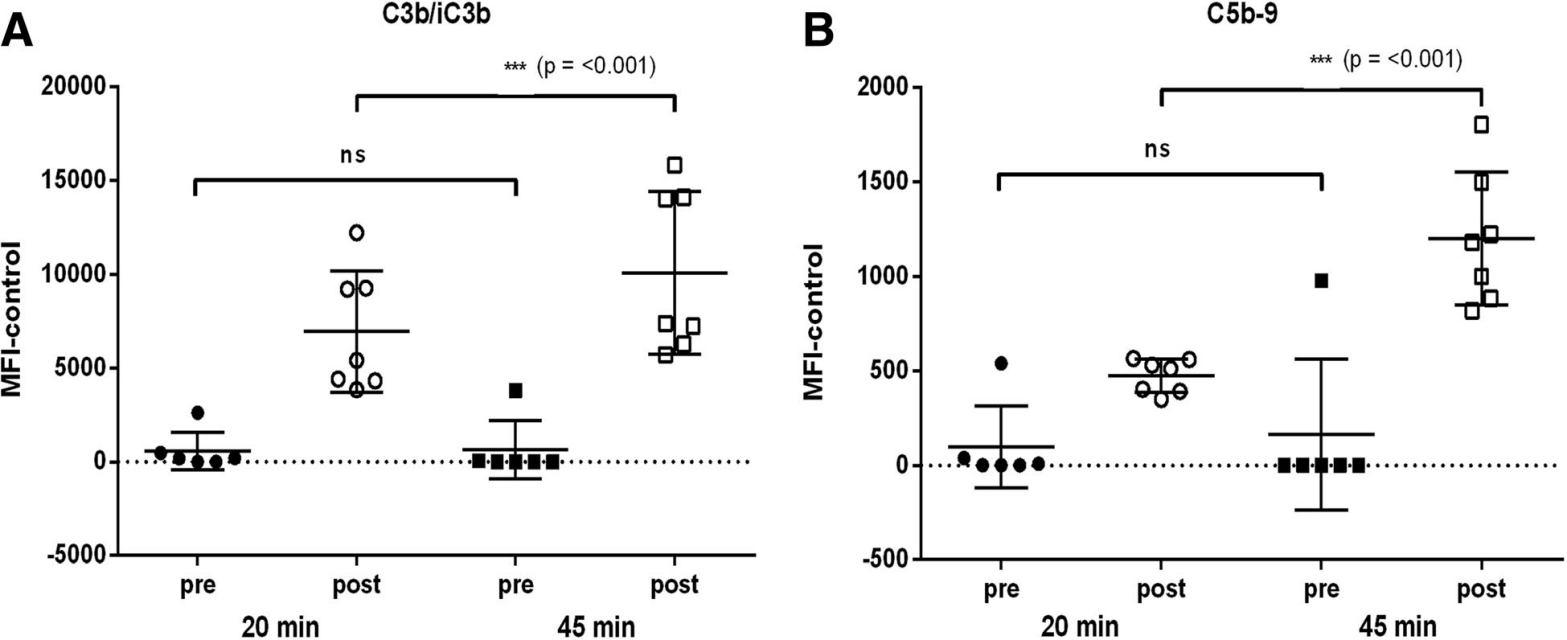
Selection of incubation time for CDA. NTHi strain 3655 was incubated with 2% IgG-depleted human plasma along with human pre and post *Synflorix* sera. Antibody-dependent (**a**) C3b/iC3b and (**b**) C5b-9 binding was determined by flow cytometry and expressed as MFI-control following incubation for 20 or 45 min. Significance was determined by two tailed t-test

**Fig. 4:**
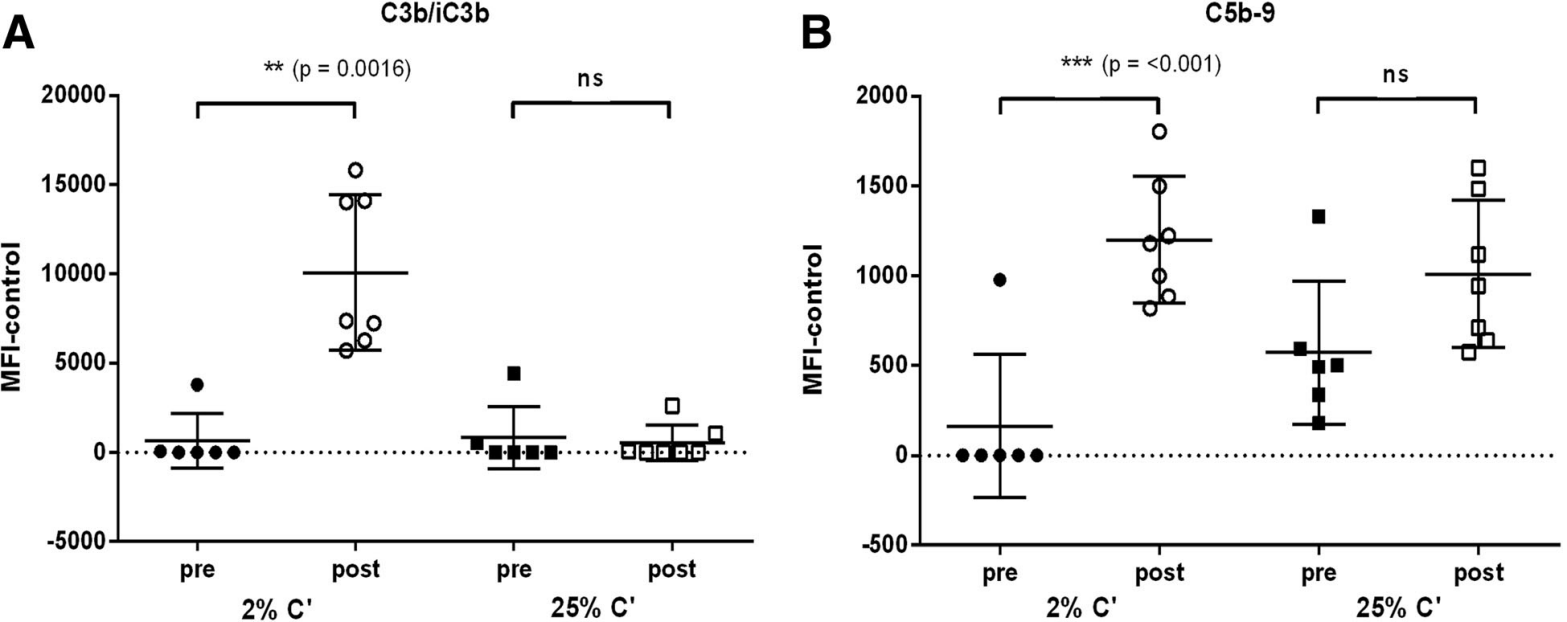
Selection of complement concentration for CDA. NTHi strain 3655 was incubated for 45 min with 2% or 25% IgG-depleted plasma in the presence of human pre and post human *Synflorix* sera. Antibody-dependent (**a**) C3b/iC3b and (**b**) C5b-9 binding was determined by flow cytometry. Significance values were determined by two tailed t-test

**Fig. 5:**
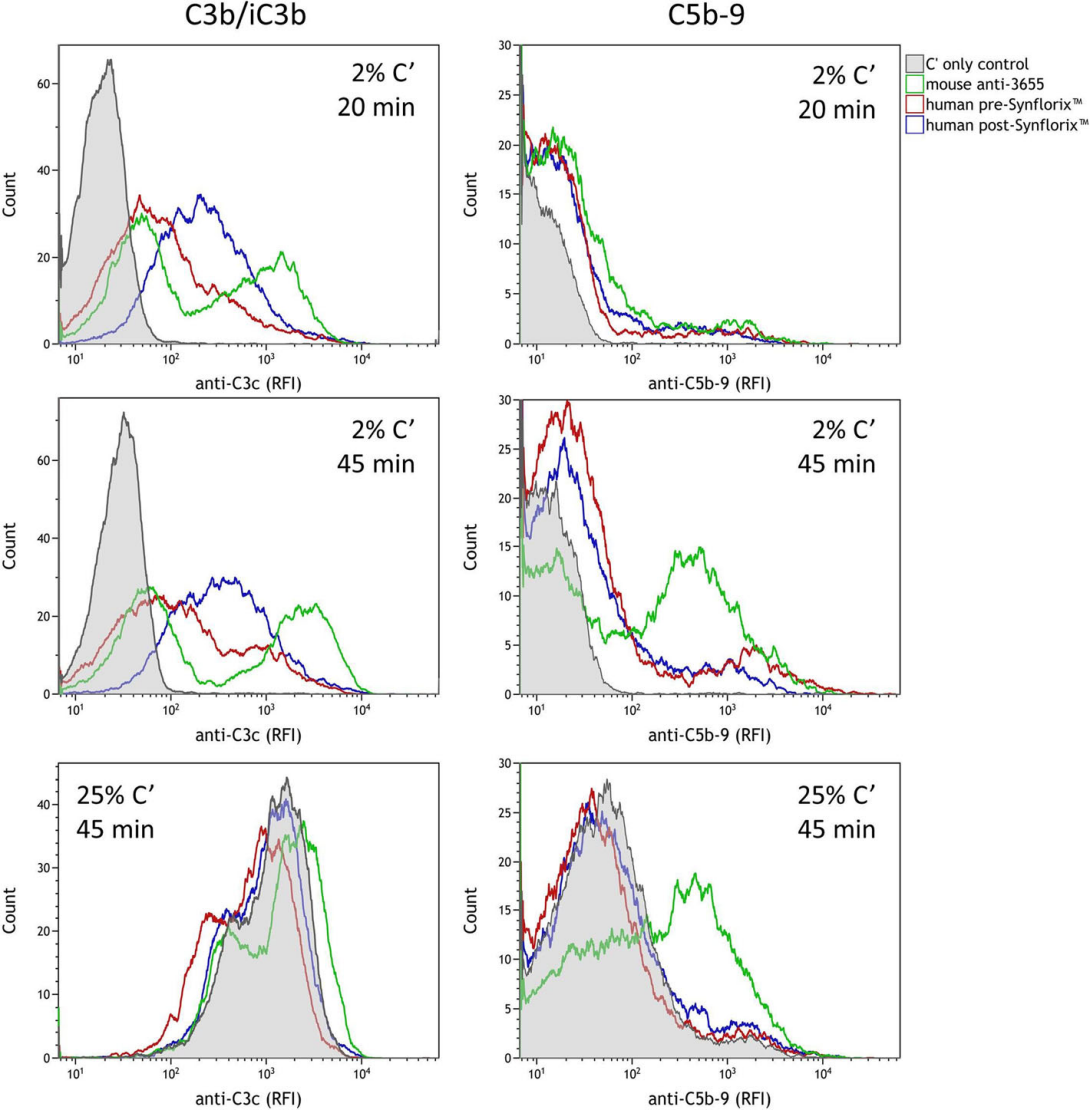
Effect of complement concentration and incubation time on antibody-independent and antibody-dependent C3b/iC3b and C5b-9 binding to NTHi 3655. Flow cytometry histograms obtained with complement-only control, mouse anti-3655 whole bacteria, or matched human pre and post *Synflorix* serum are shown. Histograms show relative fluorescence intensity (RFI) for anti-C3c (binding to C3b and iC3b) and anti-C5b-9

The complement deposition assay (CDA) was optimised for use with NTHi by adjusting the concentration of IgG-depleted plasma and the incubation times with initial conditions based on those used by Martino et al. [30] and by Ercoli et al. [16]. The effect of 2%, 5% and 25% (*v*/v) IgG-depleted plasma with incubation times of 20 and 45 min was investigated. The mean fluorescence index minus the antibody-independent control (MFI-control) values demonstrated optimum assay conditions of 2% IgG-depleted plasma with an incubation time of 45 min. MFI-control values for 20 min incubation were low while 45 min incubation resulted in greater C3b/iC3b and C5b-9 binding with good differentiation between the pre and post *Synflorix* test sera (*p* = < 0.001) (Fig. 3). The use of 25% complement in the assay resulted in antibody-independent fluorescent peaks comparable to those obtained in the presence of antibody. C3b/iC3b deposition with 2% complement showed clear differentiation between values obtained with pre and post sera (*p* = 0.0016) (Fig. 4). Although antibody-mediated C5b-9 deposition was observed for the pre and post *Synflorix* sera with 25% complement, there was no significant difference between the samples, while using 2% complement gave a significant difference (*p* = < 0.001) (Fig. 4). Overlay plots of the relative fluorescence intensity (RFI) for anti-C3c (measuring C3b/iC3b deposition) and anti-C5b-9 confirmed the effect of the increased fluorescence peak shift at 45 min compared to 20 min with the antibody-independent complement binding with 25% IgG-depleted plasma (Fig. 5).

Once optimal conditions had been established, intra-assay, inter-assay and inter-operator precision of the CDA were determined for the three NTHi strains 3655, 3224A and MPJ003 using 14 human sera. The inter-operator precision was performed by three operators on one day; the intra-assay precision was performed by one operator on the same day three times and the inter-assay precision was performed by one operator over three days. Low coefficient of variance (CV) values for all assays were obtained with the majority of samples giving a CV of < 20% for C3b/iC3b deposition (Table 2) and C5b-9 deposition (Table 3).

**Table 2:** C3b/iC3b assay variability for strains 3655, 3224A and MPJ003 (14 sera per strain in triplicate, *n* = 126)

	Description	Sera with defined CV				Mean CV (±SEM)
		< 20%	< 35%	< 40%	> 40%	
Intra-assay variability *n* = 126	3 plates, 1 operator, 1 day	34	4	1	3	13.09 (2.27)
Inter-assay variability *n* = 126	1 plate, 1 operator, 3 days	33	7	2	0	11.40 (1.55)
Inter-operator variability *n* = 126	1 plate, 3 operators, 1 day	26	6	3	7	21.47 (2.67)

**Table 3:** C5b-9 assay variability for strains 3655, 3224A and MPJ003 (14 sera per strain in triplicate, *n* = 126)

	Description	Sera with defined CV				Mean CV (±SEM)
		< 20%	< 35%	< 40%	> 40%	
Intra-assay variability *n* = 126	3 plates, 1 operator, 1 day	37	3	0	2	12.69 (1.84)
Inter-assay variability *n* = 126	1 plate, 1 operator, 3 days	30	9	1	2	15.87 (1.71)
Inter-operator variability *n* = 126	1 plate, 3 operators, 1 day	26	11	0	5	20.27 (2.60)

A correlation between C3b/iC3b deposition and OPA uptake or OPA oxidative burst was performed using a panel of 13 human sera (convenience samples previously collected from healthy laboratory staff) and produced values of 0.69 (*p* = 0.0089) and 0.65 (*p* = 0.0131) by Pearson correlation coefficient, both of which were significant. It was also observed that serum samples that had the highest fOPA also had the highest C3b/iC3b deposition.

**Fig. 6:**
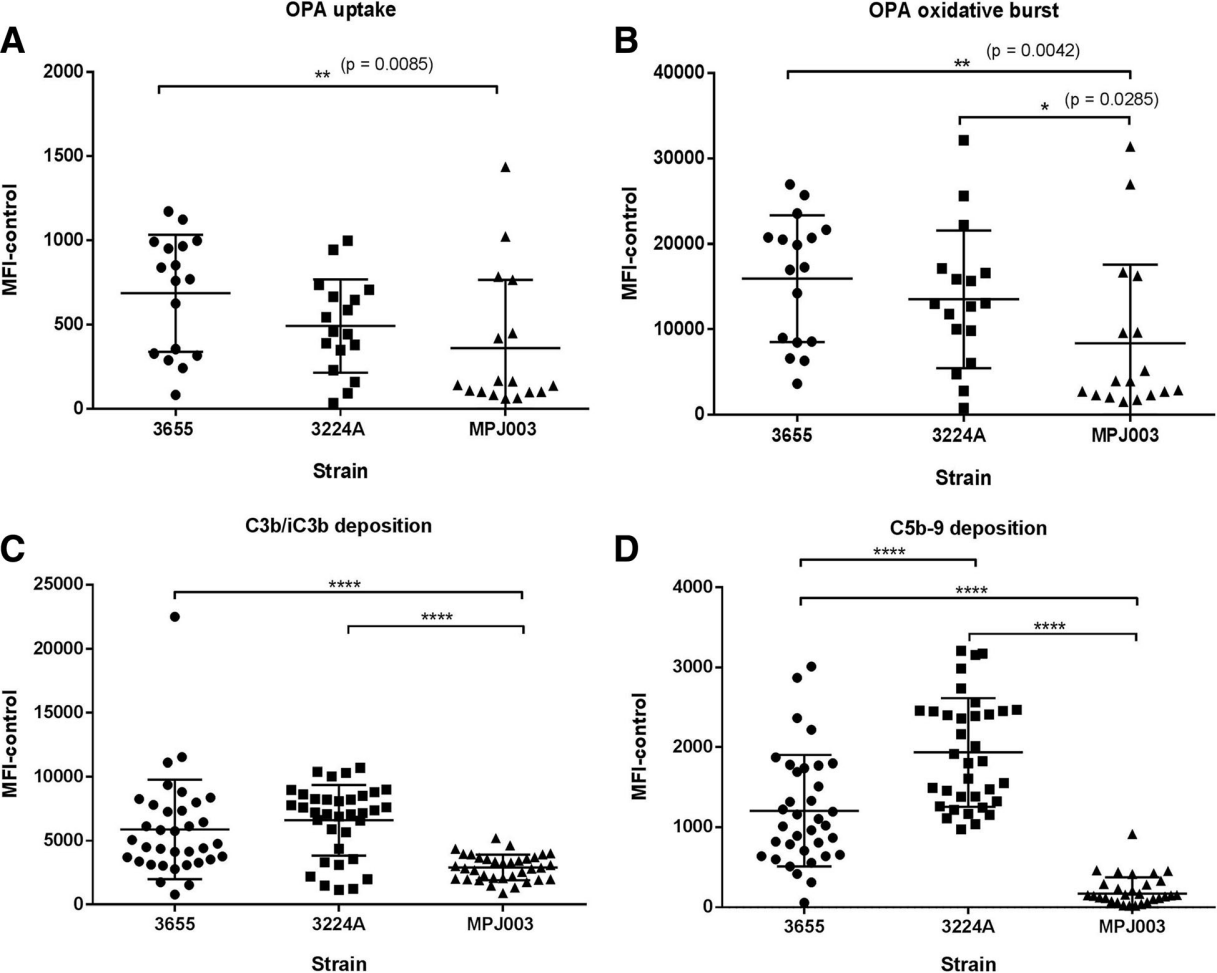
NTHi strains differ in their interaction with antibody and complement. Significant differences were observed for (**a**) OPA uptake and (**b**) OPA oxidative burst as well as for (**c**) C3b/iC3b and (**d**) C5b-9 deposition with NTHi strains 3655 (circles), 3224A (squares) and MPJ003 (triangles) in the presence of human donor serum from subjects with stable mild and moderate COPD. Significance values were determined by two tailed Mann-Whitney test (**** *p* = < 0.0001, all other significances are indicated)

Interestingly, during assay development for both flow cytometric assays, differences were observed between NTHi strains for duplexed OPA uptake and oxidative burst and for deposition of C3b/iC3b and C5b-9 using a panel of human sera from subjects with stable mild or moderate COPD (Fig. 6). Data for C3b/iC3b binding showed no significant difference between strains 3655 and 3224A, however there was a significant difference in deposition between 3655 and 3224A compared with MPJ003 (*p* = < 0.0001). Significant differences were observed between all strains for C5b-9 deposition (*p* = < 0.0001). The correlations between C3b/iC3b and C5b-9 for strains 3655, 3224A and MPJ003 were 0.38 (*p* = 0.025), 0.24 (*p* = 0.163) and 0.16 (*p* = 0.372) respectively by Pearson correlation coefficient. There appeared to be no strong relationship between those sera that had high deposition of C3b/iC3b and those that had high levels of C5b-9 deposition. As with C3b/iC3b, OPA oxidative burst showed significant differences between 3655 and 3224A compared with MPJ003, but only between 3655 and MPJ003 with OPA uptake.

## Discussion

The development of OP assays for unencapsulated Gram negative pathogens is difficult, as killing assays will measure a combination of antibody and complement-mediated killing and killing by the phagocytic cells. Although previous studies have shown that the bactericidal activity can be blocked by the addition of antibodies against late complement components [29, 31, 32]. The use of flow cytometry to measure uptake and oxidative burst by phagocytic cells has already been used for *N. meningitidis* [25, 26]. However, for NTHi we changed several parameters from the method previously described by Findlow et al. [26]. The addition of a separate incubation step for complement binding to bacteria previously incubated with antibody [25] proved to be highly effective at increasing MFI-control values, as the pre-opsonisation step allowed increased antibody binding and opsonophagocytic uptake by HL-60 cells. We initially used IFNγ as this has previously been shown to prime cells to be more responsive to bacterial uptake or promote an oxidative burst response [33, 34]. However, for the NTHi fOPA, MFI-control values were considerably higher without IFNγ pre-treatment of HL-60 cells. BCECF-AM had been used successfully for the *N. meningitidis* OPA [25] but its use with NTHi appeared to inhibit opsonisation. As a result, CellTrace violet was investigated as an alternative. This stain enters cells by diffusion through the plasma membrane and the non-fluorescent molecule is cleaved by cell esterases to produce a fluorescent molecule that is then able to covalently bind to amine groups on intracellular proteins. CellTrace violet staining proved to be very stable and it allowed the uptake OPA to be duplexed with the measurement of DHR-123 oxidative burst fluorescence. Inter-assay precision of the duplexed assay showed reproducible results, with the majority of samples giving a CV < 20%.

High levels of complement deposition were observed in the absence of antibody using CDA conditions optimised for *N. meningitidis,* [30]. Thus, for unencapsulated NTHi, optimisation was required to distinguish antibody-dependent from antibody-independent complement binding. To determine antibody-dependent complement deposition, we increased the incubation time from 20 to 45 min, and reduced the complement concentration from 25 to 2%. Although 5% complement, as used by Martino et al. [30] was investigated (data not shown), similar results to those obtained with 25% were observed. This could be because a key component of the alternative pathway, factor D, is still present in sufficient quantities to give high antibody-independent complement deposition [35]. Therefore the use of this concentration was excluded from consideration with NTHi. Measurement of intra-assay, inter-assay and inter-operator variability resulted in low coefficient of variance (CV) values for all individual test samples and all three NTHi strains, with the majority of samples giving a CV of < 20%. Overall, the antibody-mediated complement deposition assay demonstrated good precision with a total assay CV of 16%.

The protein D used for polysaccharide conjugation in *Synflorix* was not derived from any of the NTHi strains used in this study, therefore antibody rises observed between pre and post *Synflorix* vaccination sera are as a result of cross-reactive vaccine-derived anti-protein D antibodies. In addition, throughout the development of these assays it was noted that all three strains of NTHi varied greatly in OPA uptake and oxidative burst as well as the antibody-mediated deposition of C3b/iC3b and C5b-9, suggesting varying degrees of susceptibility to complement-mediated killing in-vivo, dependent on strain. Variation in serum resistance of NTHi strains has also been observed by Hallström et al. who compared isolates from patients with NTHi invasive disease with isolates taken from patients with NTHi associated upper respiratory tract infection [36]. Greater complement resistance has also been observed in isolates obtained from the middle ear with a modification of lipooligosaccharide (LOS) involved [37]. LOS modification has also been shown to be important for complement resistance of a nasopharyngeal isolate [38].

An important factor for development of both assays was the use of large scale (approximately 300 ml batches) human IgG-depleted plasma as the exogenous complement source. To date the functional immunological assays developed for use with NTHi, such as the SBA [16] or opsonophagocytic killing assay [17] have used other mammalian sources, such as baby rabbit, or have been prepared from individual human volunteers. The use of a large scale preparation from pooled plasma means that the variability between assays is reduced, while the use of a human source of complement will allow species-specific complement interactions to be studied. For example the P5 outer membrane protein on NTHi, has been shown to specifically bind the human complement regulatory protein factor H [39]. The specificity of P5 for human factor H could therefore result in overestimation of antibody function if other mammalian sources of complement were used, in a similar manner to that observed with meningococcal antibody and complement-dependent assays [40].

Assays that have been developed to date, including the SBA [16], require considerable resources for studies involving large numbers of samples as there is a limit to the number of samples that operators can process in a day. However the fOPA has been developed using a 96-well microtitre format and a single serum dilution, meaning a single operator can process in excess of 300 samples per day. The second factor to consider with other functional assays is the possible requirement in clinical trials for multiple tests (for example with different target strains) and therefore a need for large volumes of sera, for example the SBA can use 50-100 μl of serum per test when performed in duplicate [16, 41]. This is an important factor when considering clinical trials in infants where volumes of blood samples need to be kept as small as possible. An obvious advantage of the SBA is the ability to quantify post vaccination antibody rises. However, a similar quantification could also be achieved for the flow cytometric assays with the use of commercially available secondary antibody quantification beads. As mentioned, while SBA has been used in efficacy studies for Hib vaccines and shown to be a correlate of protection for invasive disease caused by Hib [18], an immune correlate of protection against disease due to NTHi has yet to be demonstrated [19]. In addition, previous studies have shown that bactericidal activity toward heterologous strains of NTHi is poor at best [3, 22, 23] and there is diversity between the methods used to measure bactericidal activity in these studies. These contributing factors have meant that this study has not correlated SBA to the flow cytometric assays developed here, but should be considered in future studies. Development of a fOPA could increase the capability of vaccine assessment by measuring both bacterial uptake and oxidative burst functional antibody activity against NTHi. A flow-cytometry based CDA to measure binding of complement components C3b (and its inactive form iC3b) and C5b-9 could provide a surrogate for the measurement of opsonophagocytosis and bactericidal activity respectively. The strong correlation between the fOPA results and antibody-mediated complement deposition shows that the complement deposition assay could be used for large scale screening of functional antibody activity against a panel of strains, for example those that represent the spectrum of complement sensitivity seen in NTHi strains. The CDA does not require the culture and differentiation of HL-60 cells and can also be performed with 5 μL of serum per test. This assay provides information on two key complement processes with C3 opsonising the bacteria and C5b-9 indicating membrane attack complex formation. The CDA also has a greater ability to differentiate antibody function against different strains making this a simple and useful tool for evaluation of anti-NTHi antibodies. Going forward, larger panels of vaccinee sera will be required (when accessible) to determine the specificity and utility of these assays for measuring vaccine-induced immunity to NTHi and the correlation with bactericidal activity should be assessed when suitable assays are available.

## Conclusions

In the absence of a recognised immunological correlate of protection for NTHi disease we have successfully developed and optimised two high-throughput flow cytometric assays which require very low volumes of serum. Both assays proved to be sensitive for the measurement of both opsonophagocytic bacterial uptake and oxidative burst using the duplexed fOPA or antibody-mediated deposition of the complement components C3b (including iC3b) and C5b-9. Both assays were shown to be highly reproducible.

NTHi is the leading cause of life threatening exacerbations of patients suffering with COPD and a key contributor to lifelong hearing complications in young children in the UK and worldwide. While a vaccine has shown efficacy against NTHi-associated AOM in children in some studies, no vaccine is currently available to prevent COPD exacerbations in adults. The flow cytometric assays developed here can contribute to an “immunological toolbox” to assist in functional immune assessment of future candidate vaccines from pre-clinical assessment, through to clinical trials and post-licensure surveillance.

## Additional files

Additional file 1: **Figure S1.** Initial measurement of opsonophagocytosis. Opsonophagocytic uptake was performed using a method previously optimised for *N. meningitidis*. High complement only backgrounds resulted in very little antibody-mediated uptake of bacteria being observed. (JPG 156 kb)

Additional file 2: **Figure S2.** Optimisation of fOPA incubation times. Incubation of bacteria, serum, IgG-depleted plasma and HL-60 cells was changed from 2 steps to 3 steps (A). Times were subsequently reduced (B). (JPG 206 kb)

Additional file 3: **Figure S3.** Selection of a fluorescent dye to measure bacterial uptake. The use of BCECF to fluorescently label NTHi showed inhibition of uptake by HL-60 cells when incubated with serially diluted human serum and IgG-depleted plasma. The Y axis is expressed as Average MFI as subtracting the antibody-independent control resulted in negative values for binding. Each point is the mean of duplicate samples. (JPG 246 kb)

Additional file 4: **Figure S4.** Relationship between opsonophagocytic uptake and oxidative burst for individual sera with NTHi, strain 3224A. Each point is the mean of duplicate samples. (JPG 484 kb)

## Abbreviations

AOM: Acute otitis media
BCECF-AM: 2′,7′-Bis(2-carboxyethyl)-5(6)-carboxyfluorescein acetoxymethyl ester
CDA: Complement deposition assay
CDA-BB: Complement deposition assay blocking buffer
COPD: Chronic obstructive pulmonary disease
CTV: CellTrace violet
CV: Coefficient of variation
DHR 123: Dihydrorhodamine 123
DMSO: Dimethyl sulphoxide
EDTA: Ethylenediaminetetraacetic acid
FITC: Fluorescein isothiocyanate
fOPA: Flow opsonophagocytosis assay
FS: Forward scatter
HBSS: Hanks balanced salt solution
Hib: *Haemophilus influenzae* type B
IFNƴ: Interferon gamma
Ig: Immunoglobulin
kOPA: Killing opsonophagocytosis assay
LOS: Lipooligosaccharide
MFI: Mean fluorescence index
NAD: Nicotinamide adenine dinucleotide
NIS: Non-immune serum
NTHi: Nontypeable *Haemophilus influenzae*
OD: Optical density
OPA-BB: Opsonophagocytosis assay blocking buffer
PBS: Phosphate buffered saline
PHE: Public Health England
SBA: Serum bactericidal assay
sBHI: Supplemented brain heart infusion broth
SS: Side scatter
