# Supplementary figures and images for: Development of flow cytometric opsonophagocytosis and antibody-mediated complement deposition assays for non-typeable Haemophilus influenzae
Source: BMC Microbiol. 2018 Oct 29;18:167. doi: 10.1186/s12866-018-1314-5 (PMC6206646; doi:10.1186/s12866-018-1314-5)

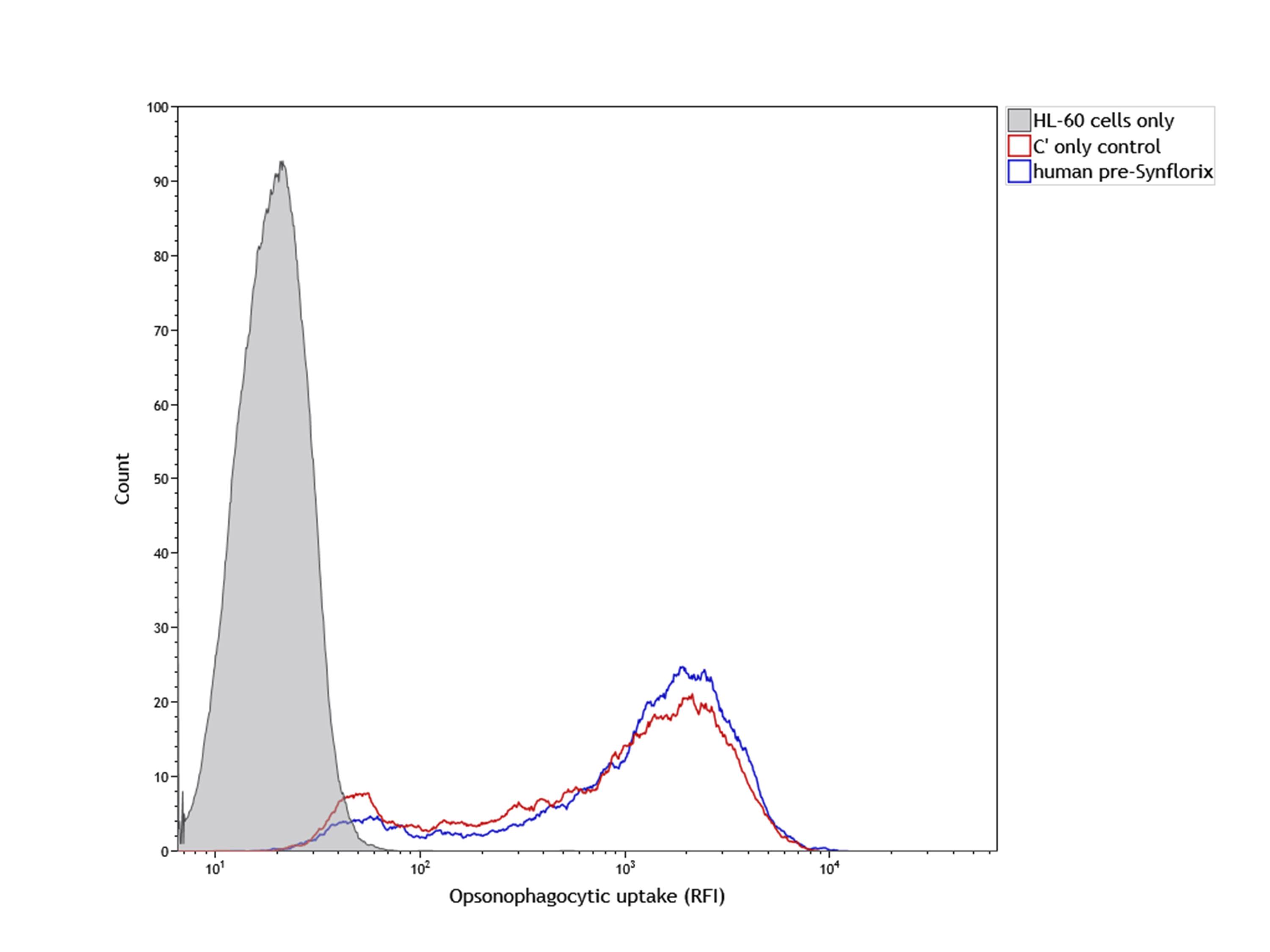

Supplement: Supplementary file 1 — Figure S1. Initial measurement of opsonophagocytosis. Opsonophagocytic uptake was performed using a method previously optimised for N. meningitidis. High complement only backgrounds resulted in very little antibody-mediated uptake of bacteria being observed. (JPG 156 kb) [file 12866_2018_1314_MOESM1_ESM.jpg]

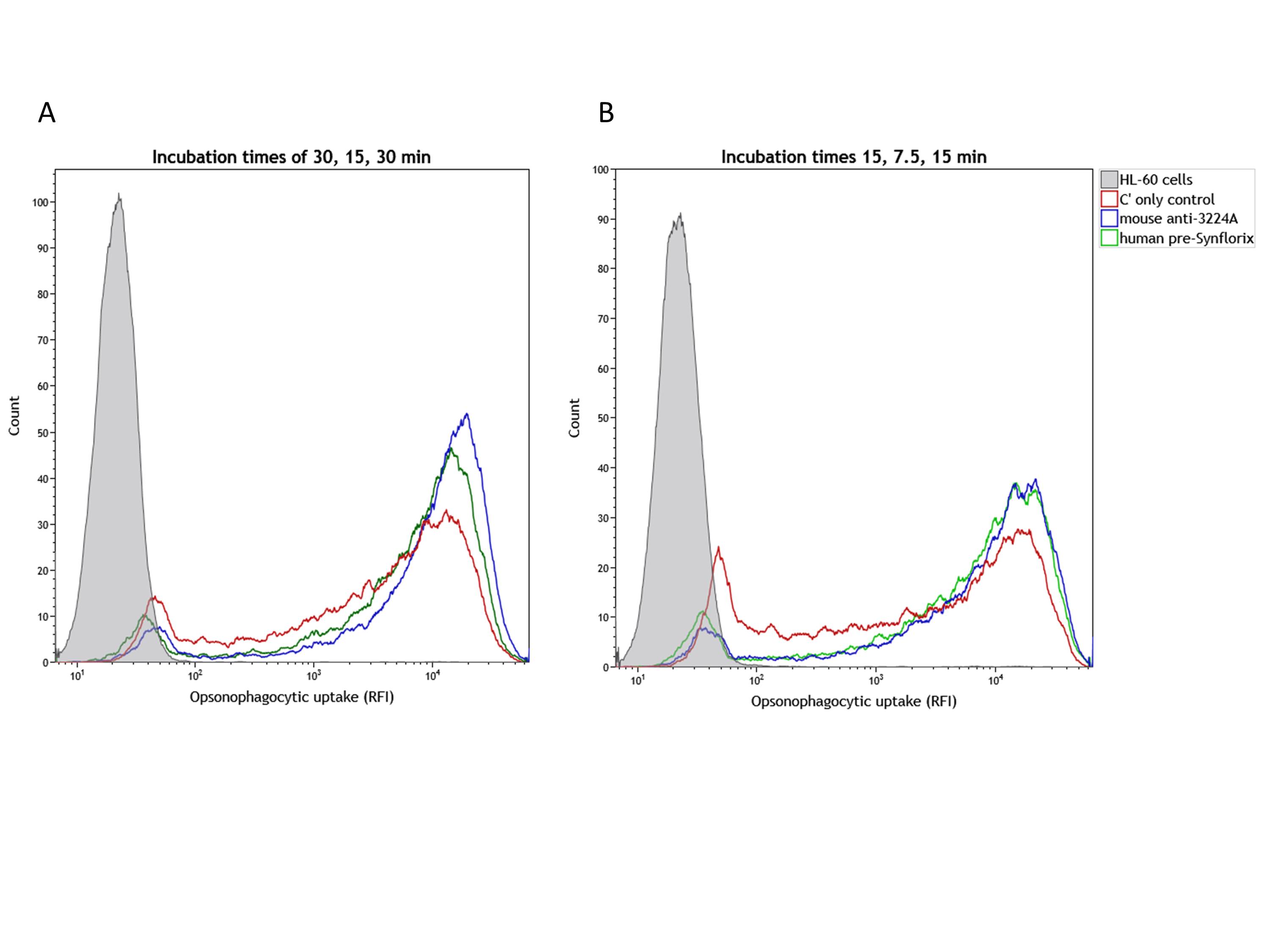

Supplement: Supplementary file 2 — Figure S2. Optimisation of fOPA incubation times. Incubation of bacteria, serum, IgG-depleted plasma and HL-60 cells was changed from 2 steps to 3 steps (A). Times were subsequently reduced (B). (JPG 206 kb) [file 12866_2018_1314_MOESM2_ESM.jpg]

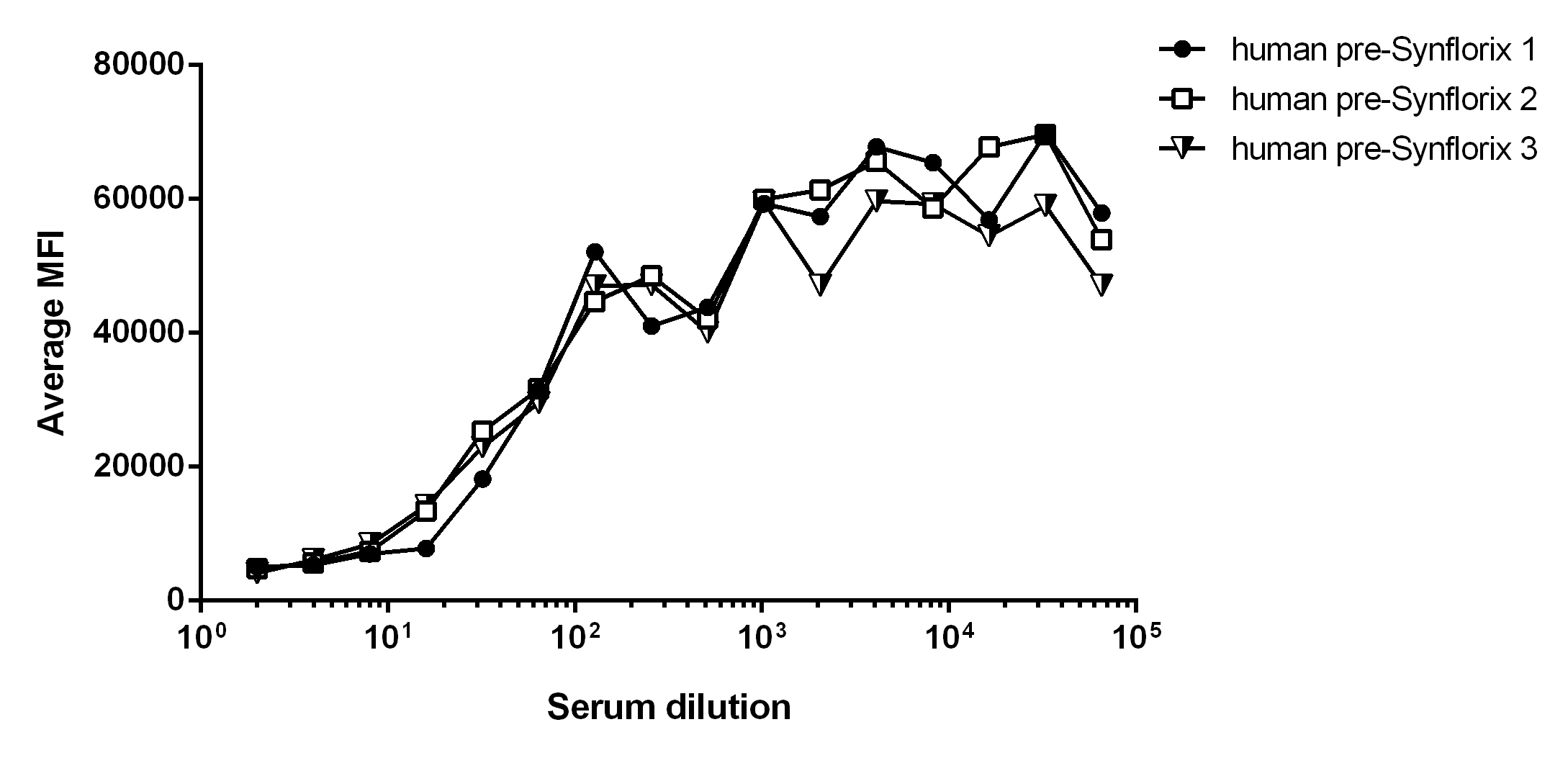

Supplement: Supplementary file 3 — Figure S3. Selection of a fluorescent dye to measure bacterial uptake. The use of BCECF to fluorescently label NTHi showed inhibition of uptake by HL-60 cells when incubated with serially diluted human serum and IgG-depleted plasma. The Y axis is expressed as Average MFI as subtracting the antibody-independent control resulted in negative values for binding. Each point is the mean of duplicate samples. (JPG 246 kb) [file 12866_2018_1314_MOESM3_ESM.jpg]

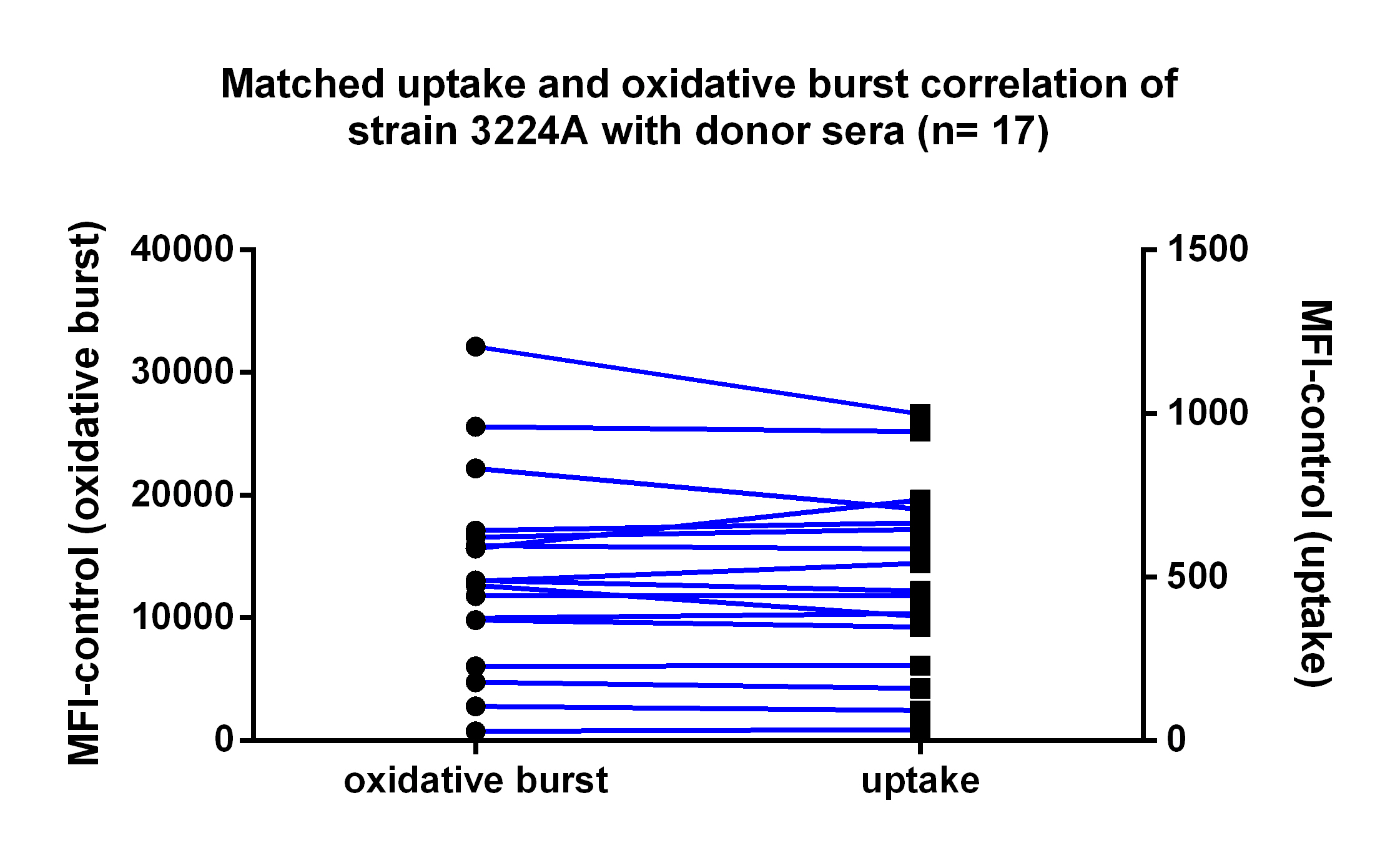

Supplement: Supplementary file 4 — Figure S4. Relationship between opsonophagocytic uptake and oxidative burst for individual sera with NTHi, strain 3224A. Each point is the mean of duplicate samples. (JPG 484 kb) [file 12866_2018_1314_MOESM4_ESM.jpg]
